# P_E_TER-assay: Combined Impedimetric Detection of Permeability (P_E_) and Resistance (TER) of Barrier-Forming Cell Layers

**DOI:** 10.1038/s41598-020-63624-1

**Published:** 2020-04-30

**Authors:** Florian Urban, Kathrin Hajek, Tobias Naber, Boris Anczykowski, Marcus Schäfer, Joachim Wegener

**Affiliations:** 1 Universitaet Regensburg, Institut fuer Analytische Chemie, Chemo- & Biosensorik, Universitaetsstr. 31, 93053 Regensburg (G), Germany; 2 nanoAnalytics GmbH, Heisenbergstr. 11, 48149 Münster (G), Germany; 3 Fraunhofer Research Institution for Microsystems and Solid State Technologies EMFT, 80686 Muenchen (G), Germany

**Keywords:** Biological techniques, Biophysics, Cell biology

## Abstract

Epithelial and endothelial barrier function is typically studied *in vitro* by growing the cells of interest on permeable supports that are sandwiched between two fluid compartments. This setup mimics the physiological situation with the cell layer as the diffusion barrier at the interface between two chemically distinct fluids. Routinely, the barrier function is quantitatively described by two key parameters: (i) the transepithelial or transendothelial electrical resistance (TER) as a measure of the permeability for small inorganic ions and (ii) the permeability coefficient (P_E_) as a descriptor of the permeability for molecular tracers. So far the two parameters have been determined in separate experiments. This study introduces a device that allows for simultaneous detection of P_E_ and TER of the very same cell monolayer in one single experiment (P_E_TER-assay). The novel approach is entirely based on AC impedance measurements in two different modes, so that TER and P_E_ become available in real time. The new approach is demonstrated for three epithelial cell lines derived from the kidney (MDCK-I, MDCK-II, NRK) with very different barrier properties under stationary conditions and when challenged by barrier-breaking fungal toxin cytochalasin D. P_E_TER provides an excellent time-resolution and completely automated data collection.

## Introduction

Solid knowledge about the structure, function and regulation of cellular barriers (e.g. skin, blood vessels, colon wall) is mandatory to understand many fundamental biomedical processes and pathologies. Cellular barriers are generally formed by monolayers of endothelial (blood vessels, lymph vessels) or epithelial cells (all other barriers). They share the expression of barrier-forming cell junctions (tight junctions) that occlude the intercellular space and avoid uncontrolled diffusion along the intercellular cleft from one side to the other^1–3^. There are three basic parameters to describe endothelial and epithelial barrier function: (i) the integral electrical resistance across the cell layer, (ii) the permeability for water and (iii) the permeability for tracer molecules^4^. *In vitro*, all three are determined by growing cells on permeable supports so that barrier tightness is accessible experimentally by (i) introducing electrodes on either side of the barrier or (ii + iii) establishing gradients of tracer compounds between both compartments. This model has been outstandingly successful and serves as a standard screening tool whenever targeting of drugs across cellular barriers or the cellular barriers themselves are addressed.

*Transepithelial or transendothelial electrical resistances* (TER in units of Ω·cm^2^) have been traditionally measured by applying low amplitude current pulses through so-called *chopstick electrodes* across the cell layer and by recording the associated voltage drop. Following Ohm’s law, the ratio of voltage to current normalized by the area provides the area-specific electrical resistance of the cell layer for small inorganic ions like Na^+^, Cl^−^, K^+^ and HCO_3_^−^. The electrodes are referred to as chopsticks because they look like a two-armed fork with one current injecting electrode and one voltage sensing electrode on either arm. The two arms are dipped into the upper and lower compartment, respectively, measuring the resistance of the monolayer. However, due to the random placement of the electrodes at the periphery of the cell layer and due to the electrode geometry, the electric field is inhomogeneous leading to systematically overestimated TER values^5,6^. Using planar current injecting electrodes that are placed in parallel to the barrier-forming monolayer has solved this problem. Recently, a new approach has been described that is based on just two instead of four electrodes: one planar electrode forms the bottom of the well, and a planar dipping electrode reaches into the upper compartment^7^. With a two electrode setup, it is mandatory to determine the TER by recording the AC impedance along an extended range of AC frequencies and by fitting the transfer function of an appropriate equivalent circuit to the experimental data. This method does not only provide the TER but also the cell layer capacitance (C_cl_ in units of µF/cm^2^) as a measure for membrane topography. Moreover, it returns the resistance of the culture medium (R_bulk_ in units of Ω) which may serve as an internal thermometer. Subtracting the impedance of an empty cell culture insert, as it is common with most other approaches, is typically not required.

A cell layer’s water permeability is typically determined by inducing an osmotic pressure gradient across the cell layer grown on the permeable support. It is expressed as the *osmotic water permeability coefficient* (P_OS_ in units of cm/s). The osmotically-induced water flux is detected from the dilution of a fluorophore in the acceptor compartment (fluorescence dilution assay^8^) or simply by the corresponding change in fluid height in a specialized glass capillary^9^. The permeability of a labelled tracer molecule is commonly quantified by establishing a concentration gradient of the tracer across the cell layer and by detecting probe accumulation in the acceptor compartment over time. The time-dependent increase of the tracer concentration gives rise to the *permeability coefficient* (P_E_ in units of cm/s). To date, mostly radio- or fluorescence-labelled probes have been used in these experiments^10,11^. When isotope labelling is applied, the permeation rate of the probe is only accessible as an *integral* descriptor of cellular barrier function. The permeation of fluorescence-labelled tracers has been followed integrally but also with spatial resolution to identify barrier heterogeneities or defects within the monolayer^12–14^. More recently, we reported the use of electrochemically active tracers for this purpose. The tracer was allowed to diffuse across the cell layer from below, while the upper surface was continuously scanned by an ultra-microelectrode to perform local quantification of probe concentration (scanning electrochemical microscopy; SECM). While permeability of the cell layer was accessible with subcellular lateral resolution, the technique suffered from low scan rates^15,16^.

This study describes a combination of two impedance-based approaches to measure TER and P_E_ of epithelial and endothelial cell layers grown on permeable supports quasi simultaneously in one experimental setup. This new device allows studying several cell layers in parallel; it is highly automated and offers an excellent time-resolution. It is based on the established setup for impedance-based determination of TER values^7^ but substitutes the single bottom electrode in the lower compartment by a co-planar arrangement of two differently sized electrodes. Whereas the dipping electrode (apical) and the larger of the two surface electrodes (basolateral) are used to record the transcellular impedance of the cell layer for TER assessment, the two co-planar electrodes in the lower compartment are used to quantify the time-dependent concentration of an electrochemically active permeability probe that was added to the upper compartment at the beginning of the experiment. Thus, the new device provides TER, the cell layer capacitance C_cl_ and the permeability coefficient P_E_ for the probe under study in one single experiment. We refer to this setup and the assay as P_E_TER which is the combination of the two established acronyms.

## Results and Discussion

### General experimental setup of the P_E_TER-device

**Figure 1:**
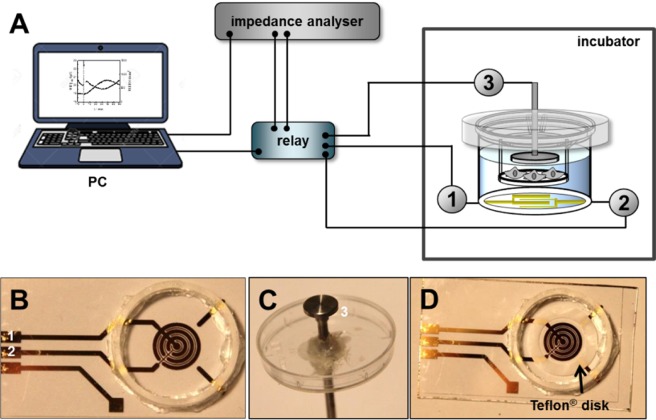
(**A**) Schematic illustration of the experimental P_E_TER-setup. The measurement chamber is stored in an incubator and a cell-covered permeable support is sandwiched between three different electrodes. A co-planar arrangement of differently sized interdigitated gold electrodes prepared on the bottom of the basolateral compartment is used for following tracer permeation across the cell layer (P_E_-mode: **1** vs. **2**). Transepithelial/endothelial electrical resistance and cell layer capacitance of the same monolayer are recorded by measuring the AC impedance between the stamp-like apical electrode dipping into the apical buffer and one of the two interdigitated electrodes (TER-mode: **1** vs. **3**). The experimental setup is completed by an impedance analyser, a relay and an ordinary PC. (**B**) Photograph of the basolateral interdigitated gold electrodes. The diameter of the chamber is 24 mm. (**C**) Photograph of the apical stainless steel electrode. (**D**) Integration of a PTFE ring into the basolateral chamber provides a well-defined receiver volume.

The experimental setup of the new approach comprises a measurement chamber, an impedance analyser, a relay and an ordinary PC (Fig. 1A) that controls the measurement and stores the data. The impedance analyser applies a sinusoidal AC voltage of a pre-set frequency and amplitude to the electrodes and detects the resulting AC current. The relay is necessary to switch between the two electrode combinations needed for reading P_E_ or TER. The measurement chamber which is the core component of the new setup is stored in an incubator to maintain physiological conditions. It contains three electrodes, two interdigitated gold-film electrodes on the bottom of the chamber and a stainless steel dipping electrode reaching into the fluid from above. The cell layer under study, grown on a standard permeable support, is sandwiched between the co-planar electrode arrangement in the basolateral compartment (1, 2, Fig. 1B) and the stamp-like dipping electrode in the apical compartment (3, Fig. 1C). This dipping electrode is mounted into the lid of a standard petri to ensure a constant working distance between electrode and the permeable support as well as to close the device and maintain sterility. The co-planar electrode structure in the basolateral compartment is composed of two differently sized interdigitated gold electrodes. The permeation of the redox tracer, added to the apical compartment at time zero, is followed by these two basolateral electrodes (P_E_-mode: 1 vs. 2) that sense the time-dependent concentration of the tracer in the basolateral fluid. TER and C_cl_ of the same cell layer are recorded by the apical stainless steel electrode and the larger of the two gold-film electrodes in the basolateral compartment (TER-mode: 1 vs. 3). A crucial requirement to extract the permeability coefficient P_E_ from the time course of tracer accumulation is exact knowledge of the volume in the receiver compartment. Therefore, a thin PTFE ring (Fig. 1D) is integrated into the basolateral chamber. Cell-covered permeable supports are placed onto the PTFE spacer creating a well-defined receiver volume.

### TER-mode measurements using the P_E_TER setup

**Figure 2:**
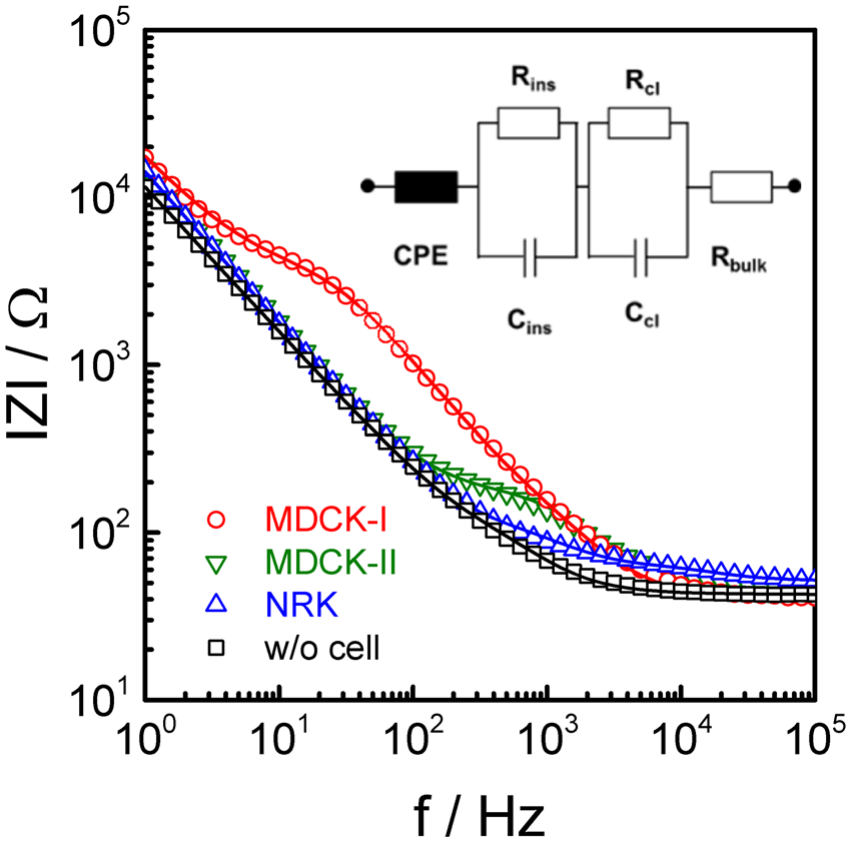
Impedance spectra recorded in TER mode (1 vs. 3) using the P_E_TER-device for confluent monolayers of MDCK-I, MDCK-II and NRK cells. The spectrum of a cell-free device is added for comparison. The raw data is analysed by equivalent circuit modelling. Least square optimization of the equivalent circuit’s transfer function returned the following parameters: (MDCK-II): R_cl_ (TER) = 88 Ωcm^2^, C_cl = _1.3 µF/cm^2^, R_bulk_ = 47 Ω, A_CPE_ = 11.4 s^n-1^µF/cm^2^, n_CPE_ = 0.94; (MDCK-I): R_cl_ (TER) = 2978 Ωcm^2^, C_cl_ = 2.0 µF/cm^2^, R_bulk_ = 41 Ω, A_CPE_ = 11.5 s^n-1^µF/cm^2^, n_CPE_ = 0.84; (NRK): R_cl_ (TER) = 11.1 Ωcm^2^, C_cl_ = 0.8 µF/cm^2^, R_bulk_ = 52 Ω, A_CPE_ = 12.0 s^n-1^µF/cm^2^, n_CPE_ = 0.90; R_ins_ = 33 Ω, C_ins_ = 4.5 µF/cm^2^.

By using the larger of the two interdigitated basolateral electrodes (**1** in Fig. 1) and the apical dipping electrode (**3** in Fig. 1), the impedance across the barrier-forming cell layer was recorded over a broad frequency range (1–10^5^ Hz). The resulting impedance spectra have been analysed with the help of an equivalent circuit that represents the electrical properties of the system under study. The corresponding equivalent circuit (inset in Fig. 2) has been developed before and is well established^7,17^. The parameters of its transfer function were fitted to the experimental data providing the electrical parameters of the system – most notably the cell layer resistance R_cl_ (=TER) and its capacitance C_cl_ (subscript *“cl”* stands for cell layer). Full description of the electrochemical system requires including (i) the electrolyte resistance of the bulk medium R_bulk_; (ii) additional impedance contributions from the setup and the permeable supports R_ins_ and C_ins_ (subscript *“ins”* stands for insert) as well as (iii) the impedance of the electrodes represented by a constant phase element (CPE) as the established electrochemical model for polarizable electrodes in physiological buffer^ 18^. Figure 2 presents typical impedance spectra recorded with the P_E_TER-device in TER-mode (**1** vs. **3**, see Fig. 1) for confluent monolayers of MDCK-I, MDCK-II and NRK cells (symbols) together with the transfer function of the equivalent circuit after least square optimization (solid line).

The frequency-dependent impedance as depicted in Fig. 2 may be divided in three frequency regimes: (i) At high frequencies (10^4^–10^5^ Hz) the impedance magnitude |Z | remains almost frequency-independent since it is dominated by R_bulk_. (ii) In the mid frequency range (10^2^–10^4^ Hz for MDCK-II, 10^3^–10^5^ Hz for NRK, 10 – 10^4^ Hz for MDCK-I) the dielectric properties of the cell layer (R_cl_, C_cl_) are reflected as a shoulder. (iii) The linear impedance increase towards low frequencies is due to the impedance of the electrode/electrolyte interface modelled as a CPE. The three cell lines are known to express barrier forming tight junctions to very different degrees that mirror their physiological origin from different sites of the collecting duct in the kidneys. NRK cells represent the class of rather leaky cell layers that are classified as *epithelial-like*. MDCK-II cells are considered moderately tight, whereas MDCK-I cells are known to build very tight barriers^19,20^. The differences in barrier function are expressed in their individual TER values ranging from 11 Ωcm^2^ for the NRK cell layer, 88 Ωcm^2^ for MDCK-II to 2978 Ωcm^2^ for MDCK-I cells. From the pool of our data we determined average TER values of (14 ± 1) Ωcm^2^ for epithelial-like NRK cells, (92 ± 4) Ωcm^2^ for MDCK-II cells and (3000 ± 130) Ωcm^2^ for MDCK-I cells. Accordingly, the order of barrier tightness NRK < MDCK-II < MDCK-I is clearly reproduced by the individual TER values which compare favourably with data taken from the literature (cf. Table 1). It is noteworthy when comparing TER values recorded in different labs for the same cell lines that they are readily affected by the external conditions and the medium composition^21^.

**Table 1:** TER and C_cl_ as recorded in this work for the epithelial cell lines MDCK-I, MDCK-II and NRK in comparison to literature studies.

cell line	TER/Ωcm^2^	Reference TER	Ccl/µFcm^-2^	Reference C_cl_
NRK	13 ± 1	^34^	0.60 ± 0.08	^41^^*#^
	12	^35^		
	12	^36^		
	14 ± 1	this work*	0.88 ± 0.04	this work*
MDCK-II	82 ± 3	^37^	1.52 ± 0.03	^41^^*#^
	136 ± 17	^38^		
	180 – 250	^39^		
	92 ± 4	this work*	1.37 ± 0.09	this work*
MDCK-I	1500	^40^	0.96 ± 0.07	^41^^*#^
	4000	^39^		
	876 ± 53	^41^		
	3000 ± 130	this work*	1.91 ± 0.06	this work*

^*^Indicates that results are given as mean ± SE. ^#^Indicates that the reported values were recorded for cells grown directly on gold-film electrodes (ECIS).

Cell layer capacitances C_cl_ are only rarely reported despite their usefulness in epithelial biology as an indicator for plasma membrane topography. The spectra in Fig. 2 reveal cell layer capacitances C_cl_ to be 0.8 µF/cm² for NRK cells, 1.3 µF/cm² for MDCK-II cells and 2.0 µF/cm² for MDCK-I cells. Pooling all experiments, the average C_cl_ values are (0.88 ± 0.04) µF/cm² for NRK, (1.37 ± 0.09) µF/cm² for MDCK-II and (1.91 ± 0.06) µF/cm² for MDCK-I cells (mean ± SE). When apical and basal membranes are entirely flat and do not show any convolutions or microvilli, the cell layer capacitance would be close 0.5 µF/cm². This value corresponds to the serial arrangement of two unfolded membranes in series, with each of the two flat membranes showing a membrane capacitance C_M_ of 1 µF/cm², as it is widely accepted. A cell-type dependent cell layer capacitance C_cl_ significantly higher than 0.5 µF/cm² reveals the expression of microvilli and other membrane convolutions that enlarge the plasma membrane surface area. The highest C_cl_ values for MDCK-I cells indicates that their plasma membrane shows the highest level of microvilli decoration, a fact that becomes important when active epithelial transport across the plasma membranes is quantitatively compared between different epithelial cell lines. Comparison to literature values is difficult as C_cl_ values are rarely reported. Table 1 compares the capacitances of NRK, MDCK-II and MDCK-I cell layers as determined in this work with C_cl_ values of the three cell lines taken from a different study from our lab^22^, when the cells were grown on planar gold-film electrodes but not on porous supports. We are well aware that cell differentiation might be different on permeable rather than non-permeable supports. We only report these values as an orientation.

Quantifying TER values by means of impedance analysis provides several striking advantages: (i) Using two planar, parallel electrodes ensures a homogenous electric field across the cell layer. Thus, the entire monolayer contributes to the recorded impedance preventing a systematic overestimation of the resistance of the cell layer, as it has been reported for chopstick electrodes^5,6^. Moreover, planar parallel electrodes in a fixed position make TER readings independent of a manual positioning of the electrodes providing significantly improved reliability and reproducibility. (ii) The P_E_TER-setup or technically similar approaches provide time-resolved TER monitoring (time resolution better than 2 min per well) without breaking the physiological temperature or humidity. (iii) Besides TER, cell layer capacitance (C_cl_), bulk resistance (R_bulk_) and constant phase element (CPE) are recorded and documented simultaneously which makes correction for cell-free permeable supports dispensable in most cases. Since R_bulk_ is strongly temperature-dependent, it provides an internal temperature control of the experiment. (iv) TER assays as performed here are highly automated and are conducted under sterile conditions. Thus, the approach ensures measurements under well-controlled external conditions.

### P_E_-mode measurements using the P_E_TER setup

**Figure 3:**
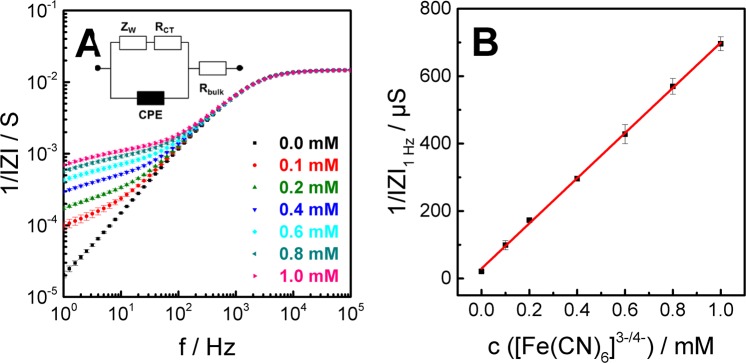
(**A**) Frequency spectra of the inverse impedance magnitude 1/|Z| in presence of increasing [Fe(CN)_6_]^3−/4−^ concentrations (P_E_-mode: **1** vs. **2**, mean ± SE, n = 3). (**B**) Increase of the inverse impedance magnitude 1/|Z | at 1 Hz with increasing [Fe(CN)_6_]^3−/4−^ concentrations (mean ± SE, n = 3). The linearity between 1/|Z|_1Hz_ and the tracer concentration is analysed by linear regression (here: slope = (670 ± 7) µS/mM; y-intercept = (29 ± 4) µS; R^2^ = 0.999).

The two co-planar interdigitated gold electrodes (**1**/**2**, cf. Fig. 1) on the bottom of the measurement chamber were used to quantify the accumulation of a redox tracer in the basolateral compartment after its permeation across the barrier forming cell layer. The electrochemical detection of the redox tracer is based on the fact that gold-film electrodes are almost ideally polarizable electrodes when bathed in physiological buffers, i.e. there is no charge-transfer across the electrode/electrolyte interface. However, when redox active species with a matching electrochemical potential are added to the bathing fluid, charge transfer across the electrode/electrolyte interface occurs. This phenomenon has been demonstrated repeatedly, for instance, when equimolar mixtures of ferri- and ferrocyanide [Fe(CN)_6_]^3−/4−^ were used as well-behaved redox species. The associated impedance change is dependent on the concentration of the species and easily measured at low AC frequencies. Accordingly, it is the rationale of the P_E_TER approach to add an electrochemically active species to the apical compartment at time zero and measure its accumulation in the basolateral compartment by impedance measurements with the help of the interdigitated gold-film electrodes integrated in the bottom of the measurement chamber. In order to determine P_E_ values, it is indispensable to establish the concentration dependence of the impedance changes that are associated with the accumulation of the probe. From impedance theory, the presence of electrochemically active species gives rise to a charge-transfer resistance R_ct_ that represents the resistance associated with electron transfer from the metal electrode to the ionic species in solution and *vice versa*. In series to R_ct_ there is the so-called Warburg impedance Z_w_ that represents the impedance associated with the diffusion-limited mass transfer of the redox species to/from the electrode. These two impedance elements, which are sometimes bundled to a so-called *faradaic impedance* Z_f_, are aligned in parallel to the constant phase element that represents the impedance of the electrode in absence of redox species. The parallel arrangement of Z_f_ and Z_CPE_ is in series (insert in Fig. 3A) to the bulk resistance R_bulk_^23–25^.

Both quantities, R_ct_ and Z_w_ are dependent on the concentration of the redox active species. To quantify both parameters individually, it is necessary to record full impedance spectra. In the interest of time resolution, we made use of an empirical approach instead that provides a linear correlation between concentration of redox active species and electrochemical measurand. As detailed in Fig. 3A for the model redox species [Fe(CN)_6_]^3−/4−^, the inverse impedance magnitude 1/|Z | at a frequency of 1 Hz changes by almost two orders of magnitude when the concentration of [Fe(CN)_6_]^3−/4−^ was increased from zero to 1.0 mM whereas 1/|Z | is hardly affected for frequencies higher than 1 kHz. This is in perfect agreement with impedance theory and experimental reports on the impedance characteristics of [Fe(CN)_6_]^3−/4−^ in physiological buffers. Plotting 1/|Z | as a function of [Fe(CN)_6_]^3−/4−^ concentration (Fig. 3B) provides a strictly linear correlation (R² = 0.999) with a slope of (670 ± 7) µS/mM. The slope of this calibration curve quantifies the sensitivity of the measurement for a given redox probe in the P_E_TER assay, provided that they prove sufficient biocompatibility and no interference with TER readings. Equimolar mixtures of [Fe(CN)_6_]^3−/4−^ were found to fulfil all requirements to serve as permeation probe in the P_E_TER assay. It is noteworthy that the charge-transfer reaction does not occur at the stainless-steel dipping electrode due to a mismatch of redox potentials for this material. Thus, the TER reading is unaffected by the presence of the electro-active species.

For the impedimetric P_E_ assay, 1 mM [Fe(CN)_6_]^3−/4−^ dissolved in the experimental buffer is added to the apical compartment. Subsequently the P_E_ probe penetrates the cell layer by passive diffusion along the concentration gradient with time. The permeation rate is dependent on the barrier function of the cell layer under study. Upon arrival in the basolateral compartment, the redox species induces a concentration dependent change of 1/|Z | at the basolateral electrodes. Using the calibration curve in Fig. 3B and exact knowledge of the receiver volume (V_R_ = 9.85 ∙ 10^−3^ cm^3^), the time-course of 1/|Z|_1Hz_ can be transformed into the time-dependent tracer accumulation in the receiver compartment n_R_(t). As detailed in the supplementary information, the molecular permeability coefficient (P_E_) is then calculated from the rate of tracer accumulation dn_R_(t)/dt which corresponds to the slope of the curve n_R_(t). Only the initial linear phase of the time course of tracer accumulation was used for analysis (i.e. linear regression) to ensure proper sink-conditions. Complete equilibration between both compartments would lead to stationary values of 1/|Z | . We have experimentally ruled out any toxicity of the tracer for the concentrations and exposure times used here (cf. fig. SI1 in supplementary information) by biochemical cell viability assays (PrestoBlue™). This electrochemical P_E_ assay was conducted for the same three cell lines that have been studied for their individual TER values above: NRK, MDCK-II and MDCK-I. Table 2 summarizes the individual P_E_ values that were corrected for the P_E_ of the cell-free permeable support (cf. materials & methods).

**Table 2:** Permeability coefficients P_E_ for the cell lines under study using [Fe(CN)_6_]^3−/4−^ as a permeability probe (mean ± standard error, n = 3).

cell line	P_E_/10^−6^ cm/s	
	impedimetric	optical
NRK	710 ± 35	19 ± 8
MDCK-II	4.2 ± 0.5	1 ± 3
MDCK-I	0.9 ± 0.2	1 ± 3

The results of the impedance-based P_E_-assay are compared to P_E_ values obtained by measuring the [Fe(CN)_6_]^3−/4−^ permeation rate by UV/VIS spectrophotometry as independent validation.

The order of decreasing P_E_ values NRK» MDCK-II > MDCK-I mirrors the tightness of the cell layers for the permeation of [Fe(CN)_6_]^3−/4−^ and corresponds to the order of increasing TER values. The lowest tracer permeability is observed for MDCK-I cells which form extremely tight monolayers as already indicated by their TER values. A slightly higher P_E_ value is obtained for MDCK-II cells that build moderate barriers. NRK monolayers grown on permeable supports are very leaky due to the lack of functional tight junctions. This low-barrier phenotype is mirrored by more than 500 times higher P_E_ values in comparison to MDCK-I layers. Since [Fe(CN)_6_]^3−/4−^ is slightly coloured, it was possible to quantify the time-dependent concentration of the probe in the basolateral compartment also by photometry at a wavelength of 405 nm as an independent control of the impedance-based approach. Optical concentration analysis returned very similar permeability coefficients of MDCK-II and MDCK-I cells that were determined to be (1 ± 3)·10^−6^ cm/s. So the outcome of optical and impedance-based concentration analysis was essentially the same. P_E_ values recorded for NRK cells were significantly smaller following optical analysis compared to the impedance-based readout. But with either readout, the P_E_ value indicates that NRK cells form very leaky epithelial barriers compared to MDCK-II and MDCK-I cells. It is noteworthy that the impedance-based P_E_ values show significantly better reproducibility as indicated by the standard error of the measurements (cf. Table 2). This is at least in part due to the fact that the impedance-based approach does not require any sampling from the basolateral compartment since the [Fe(CN)_6_]^3−/4−^ concentration is determined *in situ*. Once the redox probe is added to the apical compartment, the assay runs completely automated without any interference by the operator or opening of the incubator door. This is very different from the optical readout as performed here and throughout the literature. It requires repeated opening of the incubator and liquid handling in the lower compartment raising severe constraints to the time resolution of the optical assay. Reducing the number of data points to a minimum is the only way to limit the invasiveness of these readout approaches^26–28^ but makes the readout prone to artefacts.

To the best of our knowledge, no permeability studies using [Fe(CN)_6_]^3−/4−^ as a permeability probe have been conducted and no P_E_ values for this probe have been reported so that a direct comparison to other studies is currently impossible. However, we compared the P_E_TER-based permeability coefficients for [Fe(CN)_6_]^3−/4−^ with data recorded for other commonly used probes and found meaningful agreement that strongly supports the feasibility of the new assay (Table 3). Commonly used P_E_ probes differ with respect to their molecular mass and their charge. Whereas the dependence of P_E_-values on probe size has been systematically documented recently^10,21^, we are not aware of any study that addresses the dependence of permeation rates on the net charge of the probe even though it is very likely to play a role. In terms of mass and charge fluorescein is the probe that is closest to [Fe(CN)_6_]^3−/4−^ and the individual P_E_ values agree favourably within the limits of experimental errors. Fluorescein is supposed to pass cellular barriers predominantly across bicellular tight junctions^29^. In contrast, the paracellular permeation of macromolecules across MDCK-II layers is mainly provided by large tricellular tight junctions^30^. The question whether [Fe(CN)_6_]^3−/4−^ is transported primarily via bicellular or tricellular tight junctions cannot be addressed by the entirely integral P_E_TER assay. Besides [Fe(CN)_6_]^3−/4−^ we tested a second redox species for its performance in the P_E_TER-assay: ferrocene-methanol, or short FcMeOH. Our studies (cf. supplementary information) revealed that FcMeOH is biocompatible for concentrations used here (fig. SI1 in supplementary information) and readily detected by the basolateral electrodes following the same experimental procedures and analysis formalism as described for [Fe(CN)_6_]^3-/4^. However, there were two reasons to choose [Fe(CN)_6_]^3−/4−^ as a model species for this study: (i) The calibration curve correlating 1/|Z | with the concentration of [Fe(CN)_6_]^3−/4−^ is steeper than the corresponding curve for FcMeOH (cf. figure SI 2 in supplementary information) indicating a better sensitivity when [Fe(CN)_6_]^3−/4−^ is used as a P_E_TER probe. (ii) Due to its negative charge, [Fe(CN)_6_]^3−/4−^ will permeate across the cell layer exclusively on paracellular pathways so that P_E_ values report on the tightness of barrier forming cell junctions. In contrast, FcMeOH is suspected to migrate at least partially across the membranes due to its lipophilic and uncharged character^15^. We are currently investigating whether or not a serial application of different probes to one cell layer or the parallel application of two probes to two cell layers provide additional information.

**Table 3:** Permeability coefficients P_E_ for different permeability probes that are commonly used to characterize epithelial barrier function.

permeability probe	M/g/mol	charge	P_E_/10^−6^ cm/s	reference
10kDa-FITC-dextran	10000	0	0.13 ± 0.03	^42^
4kDa-FITC-dextran	4000	0	0.5 ± 0.1	^42^
PEG-400	900	0	4.8 ± 0.5	^42^
sucrose	342	0	0.11	^43^
mannitol	182	0	2.5	^43^
fluorescein	332	−2	5 ± 1	^42^
[Fe(CN)_6_]^3−/4−^	212	−3/-4	4.2 ± 0.5	this work

All P_E_-values have been recorded for confluent MDCK-II cells grown on permeable supports from different manufacturers.

Taken together, the impedance-based permeability measurement provides reliable P_E_ estimates that agree favourably with measurements reported by others using conventional approaches. Moreover, the P_E_ values are supported by the parallel TER readings that provide consistent characterization of the individual epithelial barrier function. The impedance-based P_E_-assay excels the conventional approaches significantly in terms of time-resolution that can be pushed well below one minute. Thus, the novel technique provides online permeability monitoring and the extraction of time course data for the permeability coefficient that has not been reported before. One limitation of the new assay at the current stage of development is the limited number of tracers that have been identified so far. Fluorescence-based assays offer a pool of permeability probes with varying molecular mass from small molecules like fluorescein to macromolecules like FITC-dextran. This set of tracers enables systematic investigations of size-dependent molecular permeability across epithelial cell layers. Such a variety of probes is not yet available or identified for the impedance-based readout but a direction of future research. Currently, the P_E_TER assay is limited to study the permeation of low molecular weight tracers. Another problem of *in vitro* permeability measurements in general is the potential impact of unstirred water layers on either side of the cell layer that build a second barrier to permeability probes and hence, lead to an underestimation of P_E_ values^31^. Mild agitation of the incubation fluid on either side of the permeable support may solve this issue but the current setup is not yet compatible with that like most established P_E_ approaches.

### Combined Impedimetric Detection of P_E_ and TER (P_E_TER-assay)

**Figure 4:**
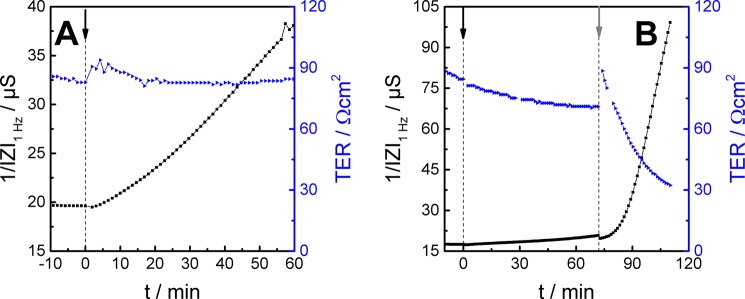
(**A**) Results of the P_E_TER-assay with a confluent monolayer of MDCK-II cells. The permeability tracer (1 mM [Fe(CN)_6_]^3−/4−^) is applied to the apical fluid compartment at t = 0 min. The accumulation of the redox tracer in the basolateral compartment is followed with time by monitoring 1/|Z|_1Hz_ (P_E_-mode: **1** vs. **2**, black). The TER-mode electrode combination is used to record the TER of the same monolayer quasi simultaneously (TER-mode: **1** vs. **3**, blue). (**B**) Confluent MDCK-II cells are apically exposed to 1 mM [Fe(CN)_6_]^3−/4−^ at t = 0 min. After 72 minutes, the barrier function is destroyed by the apical addition of 5 µM cytochalasin-D which acts as an actin polymerization inhibitor. The data in (**A**,**B**) represents individual experiments and no averages.

The parallel use of P_E_- and TER-mode measurements in what we call a *P*_*E*_*TER assay* allows characterizing the epithelial barrier function of a single cell layer with time by two independent parameters. MDCK-II cells were grown to confluence on permeable supports and mounted in the P_E_TER setup (cf. Fig. 1) which was kept in an ordinary cell culture incubator. In TER-mode impedance spectra were recorded using electrodes 1 and 3 for subsequent TER determination using equivalent circuit modelling. In P_E_-mode the impedance was only recorded at one single frequency of 1 Hz to conclude on the accumulation of the permeability probe in the basolateral compartment. At time zero the MDCK-II cell layer was exposed to 1 mM [Fe(CN)_6_]^3−/4−^ which was added to the apical fluid compartment (Fig. 4A). The time course of 1/|Z | indicates that significant permeation of the probe across the cell layer started soon after its introduction. The measured 1/|Z|_1Hz_ increases linearly by 17 µS within an observation time of 60 min. Parallel TER monitoring supports the non-invasiveness of the approach, since the electrical resistance of the cell layer remains almost constant during the entire measurement (maximum change between 82 – 93 Ωcm^2^). In a second experiment (Fig. 4B), a MDCK-II monolayer was again exposed to 1 mM [Fe(CN)_6_]^3−/4−^ at t = 0 min from the apical side and baseline data were recorded for app. 75 min. The cell layer was then challenged by 5 µM cytochalasin-D (t = 72 min). After a short delay time the presence of cytochalasin-D causes a pronounced disruption of barrier function as indicated by the massive increase of 1/|Z|_1Hz_ that amounts to almost 80 µS within 30 min. Please note the different scales for 1/|Z | _1Hz_ in Fig. 4A,B. The time-course of TER is consistent with this significant reduction of barrier function after cytochalasin-D exposure started. Initially no significant changes in TER are visible when the redox tracer is introduced into the system, but the subsequent addition of cytochalasin-D after 72 min induces an exponential decay of TER. The breakdown in barrier function is due to the well-known inhibitory effect of membrane-permeable cytochalasin-D on g-actin polymerization without affecting f-actin depolymerisation leading to net actin disassembly^32^ within several minutes. The impact of cytochalasin-D on the actin cytoskeleton has been visualised by confocal fluorescence microscopy after staining the actin cytoskeleton by fluorescence-labelled phalloidin (figure SI3 in supplementary information). Microscopic analysis confirms the pronounced perforation of the actin belt that marks the cell-cell contact zone as a continuous staining under control conditions. The results of Fig. 4 demonstrate that the new approach enables a quasi-simultaneous determination of P_E_ and TER in one experiment and within one experimental setup. This implicates a significant improvement of *in vitro* permeability studies, as the P_E_ experiments contain automatically an internal TER control as well as information on plasma membrane topography provided by the cell layer capacitance. So far permeability assays are commonly performed with TER measurements in separate setups before and/or after the P_E_ assays, mostly to ensure barrier integrity prior to the P_E_ assay. Thus, dual mode P_E_TER-assays provide a significant saving of cost and time in addition to the improved information content provided by simultaneous TER and P_E_ recordings.

## Conclusion

The novel P_E_TER-approach allows for simultaneous observation of two key parameters of epithelial and endothelial barrier function – TER and P_E_ – with a time-resolution of around one minute per cell layer. The purely electrical readout and the high level of automation require significantly less manual handling of the cell layers compared to established methods which improves reproducibility. The proof-of-concept study presented here with a maximum of two cell layers studied in parallel is scalable, providing the potential for screening for barrier function with reasonable throughput. Using multi-channel impedance meters will enable studies on epithelial and endothelial barrier function in 12-, 24-, 48- or even larger formats without sacrificing time-resolution. Feeding the calibration data into the software (e.g. by look-up tables or user input) will allow for a complete analysis of the experiment *in situ*. This study describes the use of two different redox tracers. A more extended screening for suitable permeability probes will identify further candidates and thereby broaden the information content of the measurement. It seems appealing to develop coupling chemistry to label biomolecules with electrochemically active moieties and to apply them in P_E_TER assays. A future direction of method development might look for combining different redox tracers with different permeation routes to further increase the information content of the assay.

## Material and Methods

### Cell culture

MDCK-I, MDCK-II and NRK cells were obtained from DSMZ (Deutsche Sammlung von Mikroorganismen und Zellkulturen, Germany). The cell lines were stored in a regular cell culture incubator with humidified atmosphere at 37 °C and 5% (v/v) CO_2_. Cells were grown to confluence in standard culture flasks with a growth area of 25 cm^2^ (Greiner Bio-One, Germany). Two different cell culture media were applied: (i) MEM-Eagle medium (Sigma Aldrich, Germany) containing 1 g/L D-glucose, 4 mM L-glutamine, 100 µg/mL penicillin/ streptomycin and 5% (v/v) fetal calf serum was used for MDCK-I and MDCK-II cells; (ii) DMEM (Sigma Aldrich, Germany) including 4.5 g/L D-glucose, 2 mM L-glutamine, 100 µg/mL penicillin/streptomycin and 5% (v/v) fetal calf serum was used for NRK cells. The cell culture medium was exchanged every three days. Subcultivation was conducted once a week applying a standard protocol: the cells were washed twice with PBS^–^(Sigma Aldrich, Germany), supplemented with 1 mM EDTA (ethylenediamintetraacetic acid, Merck KGaA, Germany), and subsequently treated with trypsin (0.25% (w/v) trypsin-EDTA solution (Sigma Aldrich, Germany) for MDCK-I cells and 0.025% (w/v) for MDCK-II and NRK cells. For the experiments, cells were seeded in well-defined cell densities (495000 c/cm^2^) on Transwell^®^ permeable supports (growth area: 1.12 cm^2^; polycarbonate membrane; pore diameter: 400 nm; pore density: 10^8^ pores/cm^2^; Costar Corning, LMS Consult, Germany) and stored in standard 12-well-plates (Costar Corning, LMS Consult, Germany). One day after seeding the cell culture medium was refreshed. Two days after seeding the cell-covered inserts were used for experiments.

### P_E_TER-device

The interdigitated gold-film electrodes on the bottom of the P_E_TER measurement chamber were fabricated using standard photolithographic techniques. Polycarbonate (PC, LEXAN^®^) plates were coated with a 100 nm thick, continuous gold layer (GE, Germany) by means of sputter coating (Balzers sputter coater SCD 050, Bal-Tec, Switzerland). For lithography, the gold-coated PC substrates were covered by a positive photoresist (AZ^®^-ECI3027, Microchemicals, Germany) via spin coating (model WS-650-23B, Laurell Technologies Corporation, USA) followed by a soft bake step. Subsequently the photopolymer was irradiated by UV light (isel^®^ UV illumination device nr. 2, Germany) through a shadow mask. The substrates were developed for 20 seconds with a solution of sodium hydroxide (7 g/L, Fisher Chemical, Germany) in double distilled water. The accessible gold surface was removed by a 30 sec incubation in an etching solution composed of potassium iodide (0.265 mM, Merck KGaA, Germany) and iodine (0.499 mM, Merck KGaA, Germany) under constant agitation. The etching process was stopped by a washing step with double distilled water. To get rid of the remaining photoresist, the substrate was exposed to UV light in absence of any mask and developed with sodium hydroxide as described above. A glass ring (height = 10 mm; diameter = 24 mm) was mounted on the bottom plate holding the interdigitated gold-film electrodes by a cyto-compatible silicon adhesive. The stamp-like dipping electrode was made from stainless steel. The shaft of the stamp had a thickness of 4 mm and was flattened at one end to a circular disk with a height of 1 mm and a diameter of 10 mm. In order to guarantee equal distances between the apical electrode and the permeable support as well as to close the device, the dipping electrode was integrated into a standard petri dish lid (Ø = 40 mm, TPP^®^, Sigma Aldrich, Germany). The electrode was fixed 2 mm above the permeable support.

All electrodes were electrically connected to an impedance analyser (Solartron Instruments, SI-1260, UK) via a relay that allows connecting different electrode combinations to the impedance meter. Relay and impedance analyser were controlled by a standard PC using data collection software written in LabView. The new device allowed two measuring modes with frequency settings and individual AC voltage amplitudes (rms): (i) P_E_-mode: **1** vs. **2**, 1 Hz/10 Hz, 50 mV, (ii) TER-mode: **1** vs. **3**, 1–10^5^ Hz, 10 mV. The DC potential was clamped to zero to prevent any battery effect between the two different metals used as electrodes.

### P_E_TER-assay

All impedance measurements were conducted in a standard cell culture incubator at 37 °C and 0% (v/v) CO_2_. A PBS^++^ buffer (Sigma Aldrich, Germany) containing 1 g/L D-glucose (denoted as PBS^++^/glucose (1 g/L)) served as the experimental buffer. A PTFE ring (thickness = 100 µm; inner diameter = 1.12 cm, fabricated by the machine shop at Regensburg University) was fixed to the bottom of the basolateral chamber. Cell-covered inserts were placed on the PTFE spacer creating a small cylindric buffer reservoir between the permeable support and the basolateral electrodes. This cylindric volume is referred to as *receiver volume* (V_R_) and it was calculated from its geometric dimensions to be 9.85 ∙ 10^−3^ cm^3^. The basolateral compartment was filled with 700 µL and the apical compartment with 500 µL PBS^++^/glucose (1 g/L). The redox tracer was added to the apical compartment by replacing 250 µL of the experimental buffer with 250 µL of a double-concentrated tracer solution. A 1:1 (v/v) mixture of potassium ferricyanide (K_3_[Fe(CN)_6_], Merck KGaA, Germany) and potassium ferrocyanide (K_4_[Fe(CN)_6_], Merck KGaA, Germany) in the experimental buffer was used as permeability probe and denoted as [Fe(CN)_6_]^3−/4−^. The permeability probe [Fe(CN)_6_]^3−/4−^ was applied in a final concentration of 1 mM, i.e. both complexes were present in 1 mM concentration. For some experiments, the fungal toxin cytochalasin-D (Sigma Aldrich, Germany) was added to the apical compartment in a final concentration of 5 µM.

The permeability coefficients were extracted from the experimental data recorded in P_E_-mode. Based on device calibration and knowledge of V_R_, the time-course of 1/|Z|_1Hz_ was transformed into the time-dependent accumulation of tracer in the receiver compartment n_R_(t). The linear phase of the n_R_(t) time course was analysed by linear regression providing the parameter dn_R_(t)/dt. With the other relevant parameters V_D,0_ (initial volume of the donor compartment, 0.5 cm^3^), A (area of the porous support, 1.12 cm^2^) and n_D,0_ (initial amount of tracer in the donor compartment, 5 ∙ 10^−4^ mmol) the *apparent permeability coefficient* P_app_ was calculated according to Eq. (1)^31,33^:

1 $${P}_{app}=\frac{{V}_{D,0}}{A\cdot {n}_{D,0}}\cdot \frac{d{n}_{R}(t)}{dt}$$

P_app_ was corrected for the permeability of the cell-free support (insert) P_ins_ according to Eq. (2) providing the *permeability coefficient* P_E_:

2 $$\frac{1}{{P}_{E}}=\frac{1}{{P}_{app}}-\frac{1}{{P}_{ins}}$$

The transepithelial or transendothelial electrical resistances (TER) were obtained by equivalent circuit modelling of the raw data in TER-mode. A detailed description of the modelling procedure has been reported before^7^. The applied equivalent circuit is included in Fig. 2. The transfer function of this model is fitted to the measured impedance spectra. The minimization was carried out by non-linear least square optimization (Levenberg-Marquardt). The bulk resistance (R_bulk_) represents the resistance of the bulk electrolyte and the resistive contributions arising from wiring and connectors. The constant phase element (CPE) describes the impedance of the electrode/electrolyte interface as Z_CPE_ = A^−1^(iω) ^−n^ with A and n as adjustable parameters, ω = 2πf as the angular frequency and i as the imaginary unit. The cell layer has both resistive and capacitive features which are expressed by a parallel combination of the two circuit elements R_cl_ (resistance of the cell layer) and C_cl_ (capacitance of the cell layer). R_cl_ provides the integral resistance of the cell layer which is also denoted as TER. TER and C_cl_ are normalized to the area of the permeable supports and are presented in units of Ω·cm^2^ and µF/cm^2^, respectively. The contribution of the permeable supports to the recorded impedance is considered by a parallel combination of the two parameters R_ins_ (resistance of the insert) and C_ins_ (capacitance of the insert). The values of R_ins_ and C_ins_ were determined in separate experiments and were kept constant during the fitting procedure.

## Supplementary information

Supplementary Information.
