# Supplementary material for: PETER-assay: Combined Impedimetric Detection of Permeability (PE) and Resistance (TER) of Barrier-Forming Cell Layers
Source: Sci Rep. 2020 Apr 30;10:7373. doi: 10.1038/s41598-020-63624-1 (PMC7192940; doi:10.1038/s41598-020-63624-1)
Supplement: Supplementary file 1 — Supplementary Information. [file 41598_2020_63624_MOESM1_ESM.docx]

**P_E_TER-assay: Combined Impedimetric Detection of Permeability (P_E_) and Resistance (TER) of Barrier-Forming Cell Layers**

**Florian Urban^1^, Kathrin Hajek^1^, Tobias Naber^1^, Boris Anczykowski^2^, Marcus Schäfer^2^ and Joachim Wegener^1,3,*^**

^1^ Universitaet Regensburg, Institut fuer Analytische Chemie, Chemo- & Biosensorik, Universitaetsstr. 31, 93053 Regensburg (G)

^2^ nanoAnalytics GmbH, Heisenbergstr. 11, 48149 Münster (G)

^3^ Fraunhofer Research Institution for Microsystems and Solid State Technologies EMFT, 80686 Muenchen (G)
^*^ Corresponding author: Prof. Dr. Joachim Wegener. Phone: +49-941-943-4546. 🖂 Joachim.Wegener@ur.de

**Supplementary Information**

**Testing for a potential cytotoxicity of the redox tracers [Fe(CN)_6_]^3-/4-^ and FcMeOH**

Potential cytotoxicity of the redox tracers [Fe(CN)6]^3-/4-^ and ferrocene methanol (FcMeOH) in contact to monolayers of MDCK-I, MDCK-II and NRK cells was studied via the PrestoBlue® assay that reads mitochondrial activity similar to MTT. The cell layers were incubated with 200 µL of different redox tracer concentrations (37 °C, 0 % CO_2_, 3 h). Afterwards, the solutions were substituted by 200 µL of the PrestoBlue® reagent [diluted 1:10 (v/v) in PBS^++^/glucose (1 g/L)] and incubated a second time (37 °C, 0 % CO_2_, 2 h). The fluorescence intensity of each well was measured with a 96-well plate fluorimeter (λ_ex_ = 532 nm, λ_em_ = 600 nm). [Fe(CN)_6_]^3-/4-^ was tested in a concentration range from (0 – 10) mM. In case of FcMeOH the concentration range was confined to (0 – 5) mM. Every assay included a negative control [PBS^++^/glucose (1 g/L)] and a positive control [0.5 % (v/v) Triton-X-100 in PBS^++^/glucose
(1 g/L)]. The final readout parameter is referred to as cytotoxicity index CI [in %] which is calculated from the baseline-corrected fluorescence intensity of the negative control F_0_ and the baseline-corrected fluorescence intensity of the corresponding sample F_x_:

| $CI=\left( 1-\frac{F_{x}}{F_{0}} \right)\times100\%$ | (SI 1) |
| --- | --- |

Consequently, a low CI is an indicator for negligible influences of the redox probe on mitochondrial activity. Plotting the cytotoxic index as a function of tracer concentration (figure SI 1) reveals that neither [Fe(CN)_6_]^3-/4-^ nor FcMeOH show any significant cytotoxic impact on the cell lines under study within the concentration range used here. Exposure of the cells to 1 mM [Fe(CN)_6_]^3-/4-^, as it is commonly used in P_E_TER-assays, causes negligible cytotoxic indices that are barely above baseline.


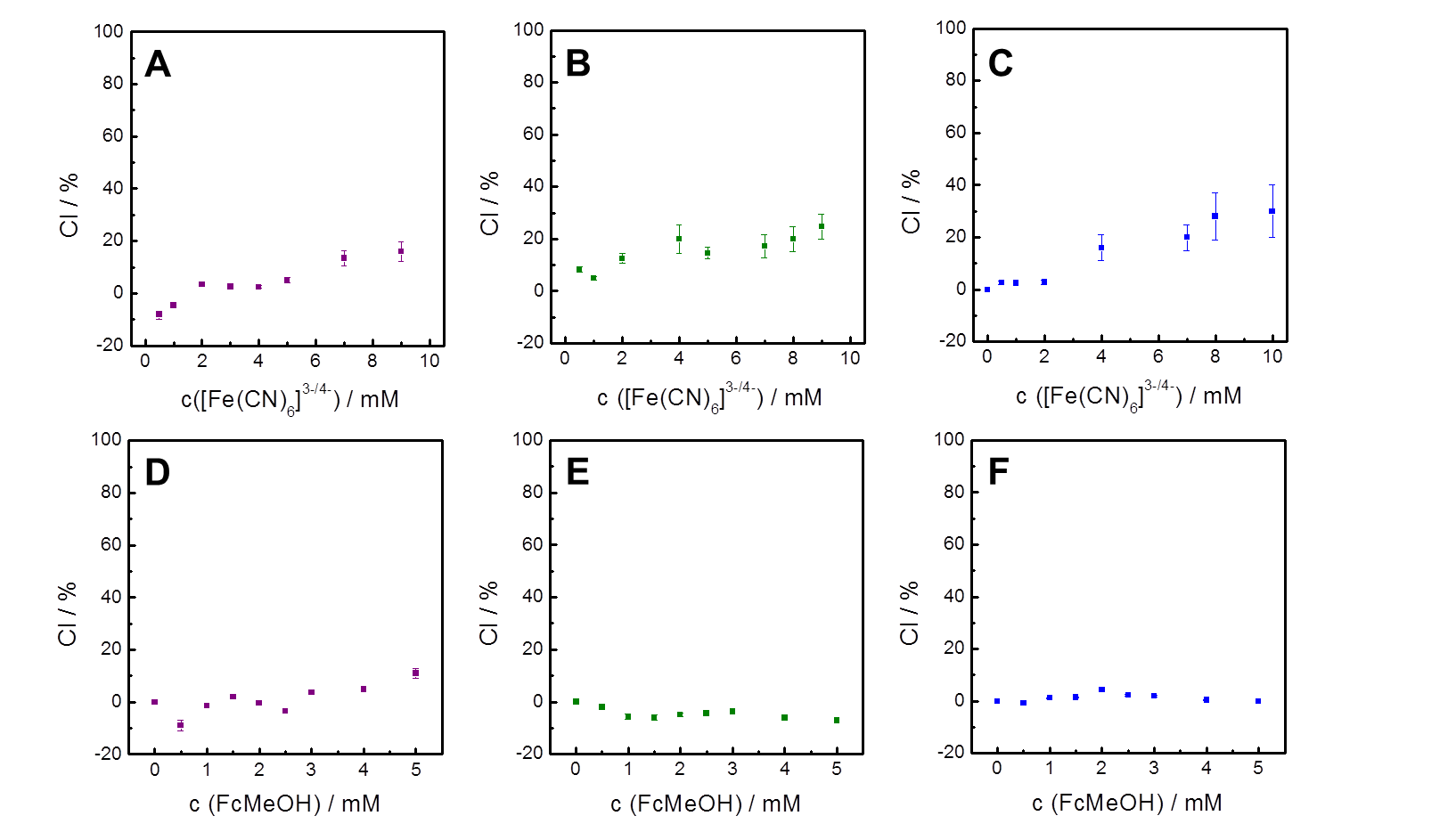


**Figure SI 1:** Evaluation of the cytotoxic potential of [Fe(CN)_6_]^3-/4-^ and FcMeOH using the PrestoBlue^®^ assays. Cytotoxicity index CI as a function of tracer concentration for (A, D) NRK, (B, E) MDCK-I and (C, F) MDCK-II (mean ± SE, n ≥ 6). Confluent cell layers were incubated for three hours with different tracer concentrations prior to assay evaluation. The results indicate no cytotoxic effects of the redox tracers within the concentration range under study. The positive controls (0.5 % (v/v) Triton-X-100 in PBS^++^/glucose (1 g/L) induced an average cytotoxic index well above 85 % in all cases.

**Comparing the sensitivity of the redox tracers in impedimetric P_E_-assays**

Different concentrations of [Fe(CN)_6_]^3-/4-^ (0 – 1 mM) and of FcMeOH (0 – 0.5 mM) were added sequentially into the chamber of a P_E_TER-device with co-planar gold film electrodes deposited on the bottom of the chamber. The complex impedance between the two coplanar electrodes was recorded over a broad frequency range (1 – 10^5^ Hz) and plotted as the frequency-dependent inverse impedance magnitude (figure SI 2). To quantify the sensitivity of the assay with the respective redox tracers, the experimental 1/IZI_1Hz_ values were plotted against the corresponding tracer concentration. The correlation between 1/IZI_1Hz_ and the tracer concentration was analysed by linear regression revealing a steeper slope for [Fe(CN)_6_]^3-/4-^ [(55.6 ± 0.9) µS/mM] compared to FcMeOH [(13.2 ±0.1) µS/mM]. A larger slope corresponds to a higher sensitivity during the impedimetric P_E_ detection. Thus, [Fe(CN)_6_]^3-/4-^ is superior to FcMeOH when used as probe in P_E_TER-assays due to an about four times higher sensitivity.


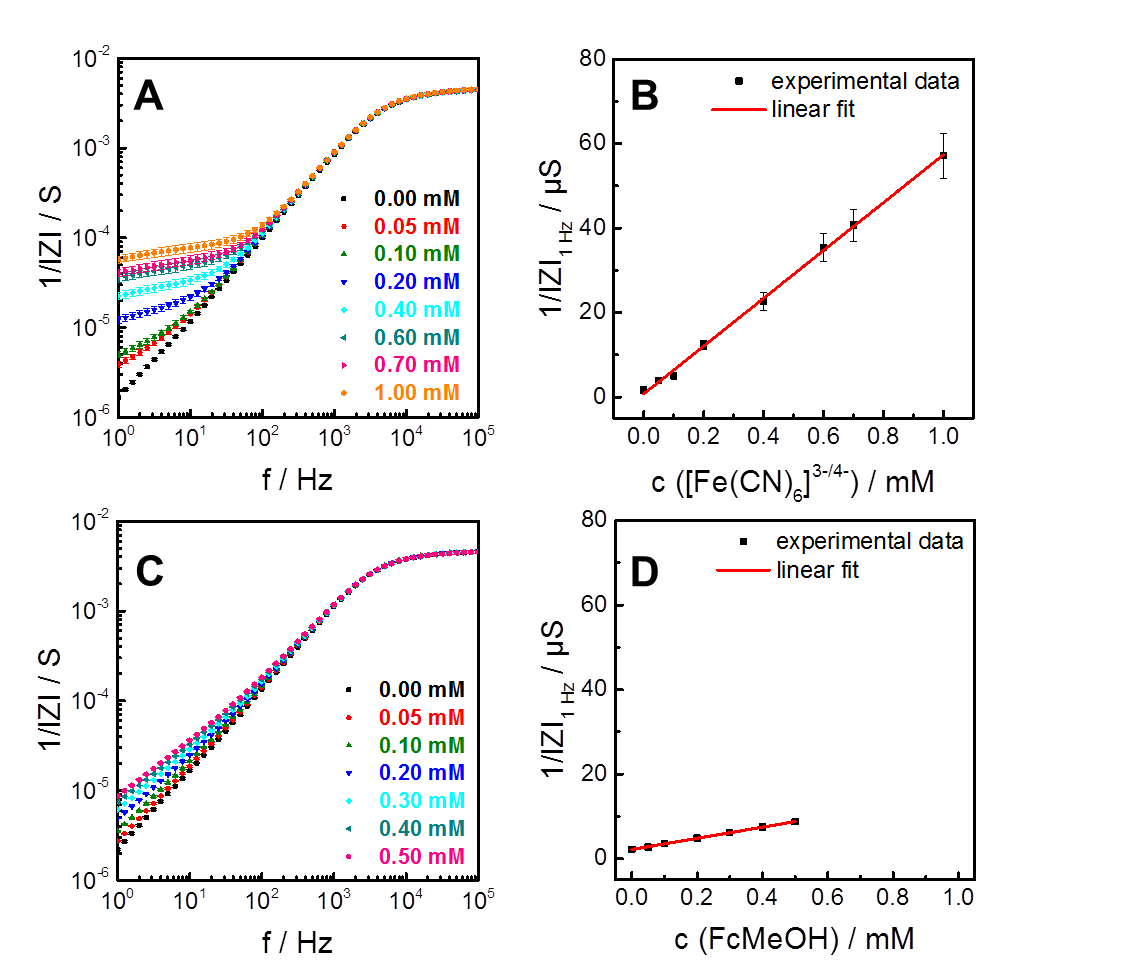


**Figure SI 2:** Comparison of the sensitivity of the P_E_TER assay when either [Fe(CN)_6_]^3-/4-^ or FcMeOH are used as redox probes in P_E_-mode (*1* vs. *2*). Frequency-dependent inverse impedance magnitude 1/|Z(f)| in presence of increasing concentrations of (A) [Fe(CN)_6_]^3-/4-^ (mean ± SE, n = 6) and (C) FcMeOH (mean ± SE, n = 6). Sensitivity is quantified from plotting 1/IZI_1Hz_ as a function of (B) [Fe(CN)_6_]^3-/4-^ (mean ± SE, n = 6) and (D) FcMeOH concentration (mean ± SE, n = 6). The experimental data was analysed by linear regression providing for [Fe(CN)_6_]^3-/4-^: slope = (55.6 ± 0.9) µS/mM, y-intercept = (0.7 ± 0.5) µS, R^2^ = 0.999 and for FcMeOH: slope = (13.2 ± 0.1) µS/mM, y-intercept = (2.18 ± 0.04) µS, R^2^ = 0.999). The slope of the linear fit-function is a measure for the sensitivity of the approach. These results indicate a more than four times higher sensitivity of the P_E_TER assay when [Fe(CN)_6_]^3-/4-^ is used as a redox probe compared to FcMeOH.

**Impact of cytochalasin-D on the barrier function of confluent monolayers of MDCK-II cells**

To illustrate the performance of the P_E_TER assay, confluent monolayers of MDCK-II cells were either treated with standard cell culture medium (control) or 5 µM cytochalasin-D (37 °C, 5 % CO_2_, 2 h). Cytochalasin-D is well known to significantly reduce epithelial and endothelial barrier function by triggering the depolymerisation of the actin cytoskeleton. Since an intact actin belt along the cell periphery stabilizes the barrier forming tight junctions, loss of cytoskeletal integrity leads to a loss of barrier function as illustrated by fluorescence microscopy after phalloidin labelling. After incubation with cytochalasin-D, the cell layers were fixed by paraformaldehyde [4 % (w/v) in PBS^++^]. Subsequently, the actin cytoskeleton was stained with Alexa Fluor^®^ 488 phalloidin (1:10 (v/v) in PBS^++^) for two hours. Unbound phalloidin was removed by several washing steps. Eventually, the sample was studied using an upright fluorescence microscope Eclipse 90i with the corresponding laser scanning unit CS1 (both Nikon GmbH). For imaging the fluorescence label was excited at 488 nm and the green fluorescence was detected at 510 nm (figure SI 3). The confocal fluorescence micrographs visualise the impact of cytochalasin-D exposure on the architecture of the actin cytoskeleton. Due to the inhibitory effect of cytochalasin-D to
g-actin polymerization the actin cytoskeleton is depolymerized and thus, barrier function of the MDCK-II layer is deteriorated.


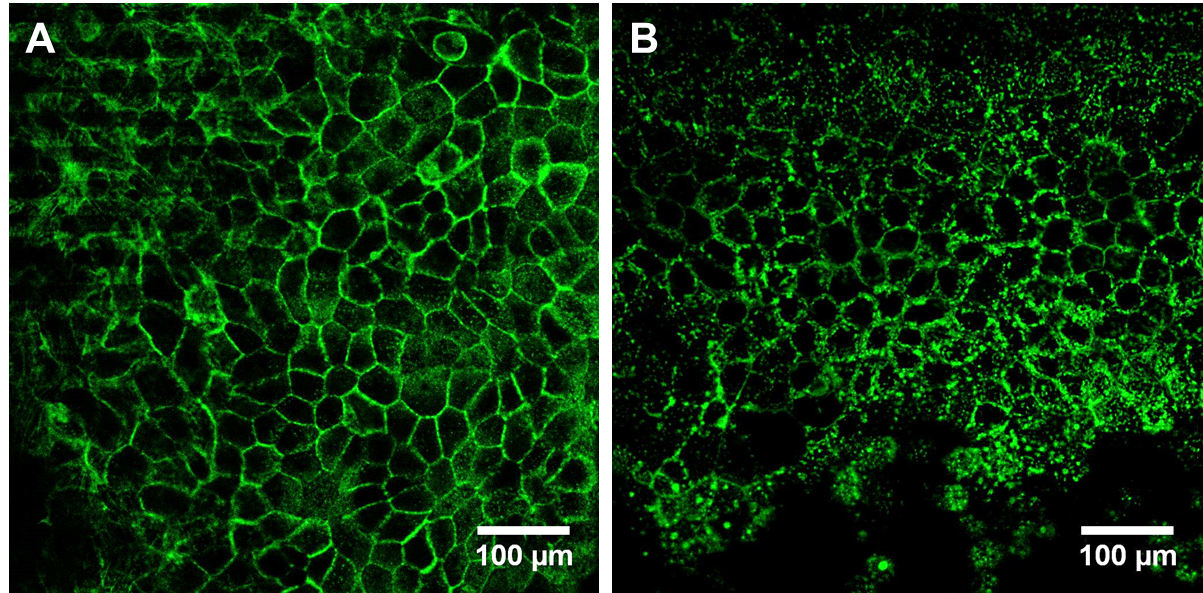


**Figure SI 3:** Confocal fluorescence micrographs of a confluent MDCK-II layer that was treated for two hours with (A) cell culture medium or (B) 5 µM cytochalasin-D. After the incubation time, the cell layer was fixed with paraformaldehyde (4 % (w/v) in PBS^++^). Subsequently, the actin cytoskeleton was stained with Alexa Fluor^®^ 488 phalloidin (1:10 (v/v) in PBS^++^) for two hours. For imaging the dye was excited at 488 nm and the green fluorescence was detected at 510 nm using a 60x water immersion objective. Micrographs visualise the impact of cytochalasin-D on the architecture of the actin cytoskeleton.
